# A Genome‐Wide Analysis of Structure and Evolution in Irish and British Populations of *Bombus terrestris* (L. 1758): Implications for Genetic Resource Conservation

**DOI:** 10.1111/eva.70141

**Published:** 2025-08-08

**Authors:** Sarah J. Larragy, Thomas J. Colgan, Eckart Stolle, Christopher Mayack, Ina Köhler, Jane C. Stout, James C. Carolan

**Affiliations:** ^1^ Department of Biology Maynooth University Maynooth County Kildare Ireland; ^2^ Botany, School of Natural Sciences Trinity College Dublin, the University of Dublin Dublin Ireland; ^3^ Institute for Organismic and Molecular Evolutionary Biology Johannes Gutenberg University Mainz Mainz Germany; ^4^ Insect Comparative Genomics Zoological Research Museum Alexander Koenig (ZFMK) Bonn Germany; ^5^ USDA/ARS/WRRC Invasive Species and Pollinator Health Research Unit Davis California USA; ^6^ Molecular Biology, Genetics, and Bioengineering Faculty of Engineering and Natural Sciences, Sabancı University Istanbul Türkiye

**Keywords:** bumblebees, genomics, island biogeography, local adaptation, pollinators, population

## Abstract

Insect pollinators play vital regulatory roles within ecosystems and provide humanity with essential services that support our health, wellbeing, and economies. Despite their importance, reported declines at regional and national levels have raised concerns over the continuation of such benefits. Island pollinator populations are of particular conservation interest as they may harbor lower genetic diversity due to restricted gene flow caused by geographical barriers, which may in turn influence local selective processes. In this study, we investigated the population structure and potential targets of selection within the genomes of a bumblebee subspecies, *Bombus terrestris audax,* native to the islands of Ireland and Great Britain. In particular, we compared the genomes of wild‐caught populations from each island alongside representatives of other European subspecies and commercial imports to ascertain patterns of historical admixture. Our analysis identified a largely genetically distinct population of *B. t*. *audax* on the island of Ireland, with weak evidence of admixture. In addition, we find differential signatures of positive selection between the two island populations in genes associated with neurology and development, indicating potential local adaptation. Furthermore, we identified an extremely polymorphic region on chromosome 10 with evidence of shared haplotypes in both wild and commercial bees, which may represent long‐standing genetic variation at the continental level or potential localized admixture between wild and commercial bees. Collectively, our findings inform on the genetic distinctiveness of these island bumblebees, emphasizing the applied need to genetically characterize natural populations to ensure the conservation of genetic resources—in the context of this study, by informing risk‐assessment and management of commercial bumblebees. In addition, our study reinforces the utility of genomic approaches in the biomonitoring of isolated or regionally adapted insect pollinator populations, which will contribute towards the effective conservation of these ecologically vital organisms.

## 1 Introduction

Global biodiversity is currently facing the highest rates of loss observed in human history (IPBES 2019). Among species showing declines are pollinating insects, which are experiencing losses in both abundance and diversity across many regions in the world (Biesmeijer et al. 2006; Cameron et al. 2011; Kosior et al. 2007; Zattara and Aizen 2019). Insect pollinators are known for their critical roles in natural ecosystem functioning and food production (Goulson et al. 2015; Klein et al. 2007; Ollerton et al. 2011). Their losses are thought to be driven by habitat loss, pathogen spread, agrochemical use, and climate change (Dicks et al. 2021; Hallmann et al. 2017; Zattara and Aizen 2019). In response to these concerning trends, national and international frameworks and policies have been developed. For example, the European Union (EU) Biodiversity Strategy for 2030 has committed to reversing the decline of wild pollinating insects by 2030 (European Commission 2018). However, the conservation of important pollinators, such as bees, butterflies, and hoverflies, is currently challenged by major knowledge gaps in their biology, behavior, and the drivers of their declines (Mayer et al. 2011).

One such factor that complicates conservation efforts of insect pollinators is that species can exhibit considerable, and often cryptic or subtle, variation across their spatial range in both their genetic and phenotypic traits (Des Roches et al. 2018; Mimura et al. 2017). Indeed, for many insect pollinator species, there is a lack of information on range‐wide intraspecific variation and gene flow despite many of them occupying ranges that span across seas and landmasses (Lecocq et al. 2017; Webster et al. 2023). While many knowledge gaps remain regarding contemporary variation across pollinating insect distributions, there are several documented cases of geographic differentiation in, for example, bee populations (Huml et al. 2023; Kelemen and Rehan 2021; Lecocq et al. 2017; Lozier et al. 2011). In particular, many studies have found that island bee subpopulations exhibit genetic differentiation from mainland or other island populations (Boff et al. 2014; Estoup et al. 1996; Francisco et al. 2016; Huml et al. 2023; Lecocq et al. 2013, Lecocq, Brasero, et al. 2015; Moreira et al. 2015). This is unsurprising, considering island populations are often exemplars of local adaptation, generally having higher incidences of endemic species and subpopulations than found on continental landmasses (Whittaker and Fernández‐Palacios 2007). Furthermore, island bee populations can be vulnerable as they often have smaller effective population sizes, harbor lower genetic diversity, and suffer from increased inbreeding and drift (Boff et al. 2014). Many present‐day island populations have also been shaped by historical glaciation events, which could increase their vulnerability as isolation in, or colonization from, glacial refugia can result in reduced genetic diversity (e.g., Pedreschi et al. 2014; Pope et al. 2006). Bumblebee populations may be at particular risk as they are eusocial, haplodiploid organisms (Packer and Owen 2001). Quantifying intraspecific variation of insect pollinators, such as bees, is vastly important to understand and predict changes in the provision of ecosystem services, which contribute fundamentally to global food security and human well‐being (Des Roches et al. 2018, 2021). Moreover, evaluating the genetic structure and potential evidence for local adaptation in isolated pollinator populations is essential for tailoring effective conservation approaches for endemic populations or evolutionary significant units (ESUs; Frankham et al. 2010; Webster et al. 2023).

One widespread pollinator species whose native distribution spans the Paleo‐arctic, including many islands, is the large earth or buff‐tailed bumblebee ( *Bombus terrestris* L.). There are nine recognized subspecies across the range of *B. terrestris* , including several endemic island subspecies, several of which show distinctions on morphological, behavioral, and genetic levels (Chittka et al. 2004; de Jonghe 1986; Estoup et al. 1996; Ings et al. 2005; Ings et al. 2009; Lecocq, Brasero, et al. 2015; Lecocq, Coppée, et al. 2015; Rasmont et al. 2008). As *B. terrestris* is an abundant, generalist species, it is an important pollinator for many wild plants and crops (Corbet et al. 1993; Goulson 2003). For this reason, it has also been domesticated for commercial use by companies that rear and export captive‐bred colonies globally to assist with crop pollination (Velthuis and van Doorn 2006). Imports of commercial bumblebee colonies into Ireland numbered nearly 5000 colonies in 2024, while imports into the United Kingdom are estimated at 65,000 colonies per year (Bumblebee Conservation Trust 2016; DAFM Open Data). Additionally, *B. terrestris* is rapidly becoming one of the main model bumblebee species for both molecular and ecological studies alike (Leadbeater and Chittka 2007). Furthermore, while *B. terrestris* is a widespread and ‘least‐concern’ species (Rasmont et al. 2015), subpopulations within the distribution of ‘least‐concern’ species can experience unique threats to their conservation status (Buckley et al. 2019; Huml et al. 2023; Thakur et al. 2018). In the context of *B. terrestris* , this may be particularly true in regions importing and using commercial colonies distinctive from the local populations, which could lead to introgression of maladapted alleles or loss of locally adapted phenotypes. Additionally, the need to monitor common and/or widespread populations is vital as losing these populations may impact ecosystems more drastically than the loss of small or threatened populations (Gaston and Fuller 2008; Huml et al. 2023).

However, despite it being one of the most well‐studied species of bumblebee (Yuan et al. 2025), we still have much to learn about the genetic variation across the native range of *B. terrestris* and the consequences of commercial bumblebee use in areas within its native distribution. In this paper, we focus on wild‐caught populations of *B. terrestris* ssp. *audax*, found on the islands of Ireland and Great Britain, which both import the same stocks of commercial bumblebee colonies, also classified by suppliers as *B. t. audax*. The island of Ireland became isolated from other landmasses towards the end of the last ice age (ca 14,000–16,000 years ago), approximately 7000 years before the island of Great Britain became isolated from mainland Europe (Edwards and Brooks 2008; Shennan et al. 2000). While many species may have naturally arrived or been introduced to Ireland since its separation from Great Britain (Montgomery et al. 2014; Power et al. 2023), there is evidence that populations could have been established in Ireland prior to this, surviving via a glacial refugium in the south of the island or by being cold‐adapted (Edwards and Brooks 2008; Teacher et al. 2009). Therefore, distinctions may have arisen among Irish biodiversity due to an extended period of separation from mainland populations, in addition to exposure to location‐specific selective pressures and non‐adaptive processes. Indeed, there are many examples of distinctive fauna between the islands, as reviewed by Sleeman (2014). Genetic distinctions have also been identified between Irish and British populations of two bumblebee species, *B. monticola* and *B. pratorum* (Huml et al. 2023). One previous study has been conducted on Irish and British *B. terrestris* populations which, to our knowledge, is the first evidence of genetic divergence between *B. terrestris* on the islands of Ireland and Great Britain (Moreira et al. 2015). However, this study was based on microsatellite data which, although provides valuable insight into population structuring, has limitations when compared with whole genome sequencing (WGS) approaches (Fischer et al. 2017; Putman and Carbone 2014; Väli et al. 2008) and so the extent of genome‐wide differences in terms of genetic variation and putative targets of selection has yet to be fully examined comparatively for these populations.

In this study, we use WGS data from wild‐caught *B. t. audax populations*, sampled across the islands of Ireland (Larragy et al. 2023) and Great Britain (Colgan et al. 2022). Additionally, we sequence commercially sourced *B. t. audax*, representative of stocks currently available for use on both islands, in addition to wild‐caught continental European *B. terrestris* subspecies (*B. t. terrestris* and *B. t. dalmatinus*) which, up to a decade ago, were the non‐native subspecies available for import into Great Britain and Ireland for crop pollination purposes. Using these data, we aim to build upon the findings of Moreira et al. (2015) and evaluate whether Irish and British *B. t. audax* populations exhibit genome‐wide differentiation from one another and if they exhibit any evidence of admixture. Secondly, we evaluate whether Irish and British populations exhibit differential targets of selection. Finally, we make preliminary observations on genetic differentiation between commercial imports and wild‐caught bees in Ireland and Great Britain. We anticipate that, as a result of reduced gene flow and local adaptation, we will find genome‐wide distinctions in both genomic variants and potential regions under selection between Irish and British populations of *B. t. audax*. Similarly, given recent findings of Franchini et al. (2023) which revealed distinctions between wild British and commercial *B. t. audax*, we hypothesize that commercial bees currently being imported into Ireland will also be genetically distinct from Irish wild‐caught bees, raising questions over the appropriateness of their use in geographical regions where commercial bees are not representative of the wild population. The findings of this study will provide key insights into the intraspecific distinctions that exist across populations of *B. t. audax* that will inform conservation efforts to conserve genetic variation and therefore adaptive ability in this keystone species.

## 2 Methods

### 2.1 Sample Collection

To perform a population genomics analysis on *B. terrestris audax* sampled from Ireland and Great Britain, we used publicly available whole genome sequencing datasets from Larragy et al. (2023; *n* = 33 Irish‐caught individuals; NCBI BioProject PRJNA870415) and Colgan et al. (2022; *n* = 51 British‐caught individuals; NCBI BioProject PRJNA628944), respectively (Table S1b,c). For both datasets, sequences were derived from haploid males. In addition, we sampled the genomes of a further six wild‐caught *B. terrestris* males representing two different *B. terrestris* mainland European subspecies (*B. t. terrestris* sampled from Germany (*n* = 3; Table S1d); *B. t. dalmatinus* sampled from Türkiye (*n* = 3; Table S1d)), which were stored in 95% ethanol prior to DNA extraction. As commercial bumblebees largely represent closed populations with lower effective population sizes (Velthuis and van Doorn 2006) and are, therefore, likely experiencing stronger effects of drift, we sequenced only a small number of commercial individuals from two independent suppliers, which we refer to as Supplier 1 (*n* = 6) and Supplier 2 (*n* = 5; Table S1d) as a representative comparison group to determine if wild‐caught bees are genetically differentiated from commercial imports.

### 2.2 DNA Extraction, Library Preparation and Sequencing

We extracted genomic DNA from bumblebee heads using the DNeasy Blood and Tissue Kit (Qiagen, Valencia, CA), with certain modifications, as described in Larragy et al. (2023). For each sample, we assessed the quality and yield of extracted DNA using agarose gel electrophoresis and a NanoDrop Spectrophotometer (ThermoFisher Scientific). We sent high quality DNA to a commercial sequencing company (Novogene, Cambridge, UK) for individual PCR‐free library preparations using the NEBNext Ultra I DNA library kit before being multiplexed and sequenced (paired‐end 150 bp) on an Illumina NovaSeq6000 platform.

### 2.3 Quality Assessment of Sequencing Data, Filtering, and Alignment

We assessed the sequence quality of raw reads of both the newly sequenced samples (*n* = 17) and the publicly available datasets (*n* = 84) using FastQC (v.0.11.9; Andrews 2010). Across all samples (*n* = 101), there was a mean of 11.2 million raw reads per sample before filtering (s.d. = 5.1 million reads) with a mean estimated raw coverage of 7.9X (s.d. = 3.8X). Raw reads were of generally high quality, with 95.74% of reads having a mean Phred quality (Q) score of 20 or greater and 90.21% of reads having a mean Q score of 30 or greater.

To remove adaptor contaminants and sequences of low quality, we filtered sequences using FastP (v.0.23.2; Chen et al. 2018). More specifically, we removed Illumina adapters, sequences with more than 10% ambiguous bases, sequences that had 40% of bases with Q scores of 20 or less, and sequences that were less than 50 bases long post‐filtering. For each sample, filtered reads were aligned against the most recent *B. terrestris* reference genome assembly (iyBomTerr1.2, GCA_910591885.1) using bwa‐mem2 (v.2.0pre2; Vasimuddin et al. 2019), with subsequent compressing and sorting of aligned reads performed by SAMtools (v1.16.1; Li et al. 2009) and duplicate marking performed by samblaster (v.0.1.26; Faust and Hall 2014).

### 2.4 Variant Calling and Filtering of Single Nucleotide Polymorphisms

Using all samples, we used freebayes (v.1.3.6; Garrison and Marth 2012) to call variants from the resulting alignment files using the parameters: ‐‐ploidy 2 –report‐genotype‐likelihood‐max ‐use‐mapping‐quality –genotype‐qualities –use‐best‐n‐alleles 4 –haplotype‐length 0 –min‐base‐quality 3 –min‐mapping‐quality 1 –min‐alternate‐frac 0.25 –min‐coverage 1. We filtered calls using VCFtools (v.0.1.17; Danecek et al. 2011) to remove insertions and deletions (“indels”), as well as sites that had quality (QUAL) scores of less than 20 or a maximum mean depth of more than 100 bp. While males are hemizygous, we performed calls with individuals marked as diploids to check for the proportion of heterozygous sites, which likely represent technical artefacts or the potential for a true diploid male, which can naturally occur. Using this approach, we removed one *B. t. dalmatinus* individual. To reduce the complexity of our dataset, we restricted our analysis to biallelic SNPs found in at least two individuals, which were identified on the 18 linkage groups outlined in the *B. terrestris* BomTerr1.2 reference genome assembly. Using these filtered calls, we next calculated the percentage of missing calls for each individual using VCFtools (parameter ‐imiss). This analysis identified variation in missing sites across samples; to maximize the number of samples used for our analysis, we created two datasets: (1) for examining population structure and patterns of admixture, we created a dataset containing only samples (*n* = 89) with less than 15% missing calls; and (2) for examining signatures of recent selection, we created a reduced dataset consisting of samples (*n* = 75) with less than 10% missing calls. For each dataset, we then removed known related individuals from the commercial colonies, and then filtered to retain calls found in all individuals (‐max‐missing 1.0). Using this approach, our first dataset consisted of 536,466 SNPs found in 89 individuals, while the second dataset contained 1,131,226 SNPs identified in 75 individuals. Using this second dataset, for bees sampled from Ireland and Great Britain, we individually estimated genome‐wide means of nucleotide diversity (π) for each island population using 10 kb sliding windows performed by Pixy (v.1.2.5; Korunes and Samuk 2021) and compared differences in genome‐wide means using a Wilcoxon rank‐sum test. SNPs were annotated with SnpEff (v.4; Cingolani et al. 2012) using gene annotation information from NCBI *Bombus terrestris* Annotation Release 103.

### 2.5 Population Structure and Genetic Differentiation‐Based Analyses

To assess the population structure in our first dataset, we carried out two main analyses on pruned SNPs. By using pruned SNPs, we reduce the number of SNPs in our analyses that are in linkage disequilibrium. Using these data, a principal component analysis (PCA) was performed (parameters: ld_threshold: 0.1, max_slide_bp: 100000; max_slide_snp = 1000) using the R package SNPrelate (v1.30.1; Zheng et al. 2020). Second, using the same pruned SNPs, an ADMIXTURE‐based (v.1.3.0; Alexander et al. 2009) analysis was performed. To calculate the most likely *K* value for our dataset, ADMIXTURE was performed for *K* = 1–20 with cross‐validation (‐cv). Each iteration for each value of K was repeated eight times. We visualized ADMIXTURE‐produced clusters using pong (v.1.5; Behr et al. 2016).

To further determine genetic differentiation between putative populations on the islands of Ireland and Great Britain, we calculated the number of polymorphic sites, mean dxy (i.e., the average number of nucleotide differences between populations per site) and F_ST_ values for 10 kb sliding windows across the 18 chromosomal scaffolds of the *B. terrestris* genome assembly using Pixy (v.1.2.5; Korunes and Samuk 2021). For each measure, we z‐score transformed values and calculated associated *p*‐values for outlier windows to identify windows with signatures of elevated genetic differentiation between bees collected from the geographically isolated sites.

### 2.6 Identification of Genomic Regions With Recent Signatures of Positive Selection

To assess signatures of selection acting differentially on loci between *B. terrestris* from Ireland and Great Britain, we also performed a cross‐population test for extended haplotype homozygosity (XP‐*n*SL) that compares haplotype patterns between the two groups to identify regions showing evidence of ‘hard’ or ‘soft’ selective sweeps (Szpiech et al. 2021). Using selscan (v.2.0.0; Szpiech 2024), SNPs with a minor allele frequency less than 0.05 were first filtered before XP‐*n*SL scores were calculated for each SNP. The score for each SNP was subsequently normalized against background genome‐wide estimates using selscan ‘norm’ to account for genome‐wide variation in recombination rates and demography. We employed a windows‐based analysis (10 kb windows) to estimate the mean normalized XP‐*n*SL score, as well as the proportion of XP‐*n*SL scores with evidence of recent selection (i.e., normalized |XP‐*n*SL| ≥ 2; Szpiech et al. 2021) for each window. Using these proportions, we z‐score transformed values and calculated *p* values to identify outlier regions with elevated patterns of extended haplotype homozygosity in bees sampled from Great Britain and Ireland, respectively. For the examination of shared haplotypes among samples for regions of interest, we generated genotype matrices and performed hierarchical clustering using SNPRelate. In addition, for candidate regions of interest revealed through our analysis, we further assessed a diploid dataset (*n* = 95 whole genome samples) generated by Kardum Hjort et al. (2022), a study performed in Sweden that consisted of wild‐caught and commercial *B. terrestris* workers (BioProject: PRJEB49221), to determine if such regions were conserved in other population genomic datasets. Using the same approach above, we quality assessed and filtered reads before aligning to the same reference genome assembly. We then estimated measures of dxy and F_ST_ between wild‐caught and commercial bumblebees using Pixy.

### 2.7 Gene Ontology Term Enrichment Analysis

To better understand the functional roles of genes with distinctive signatures of selection between *B. terrestris* from Ireland and Great Britain, we performed Gene Ontology (GO) term enrichment analyses on the outputs of the XP‐*n*SL analysis. As there is low resolution in GO terms assigned to *B. terrestris* genes, *Drosophila melanogaster* GO terms were first obtained from Ensembl Metazoa Biomart (Kinsella et al. 2011) and then assigned to their respective bumblebee homologues, which were also identified through Ensembl Metazoa Biomart. For each analysis, we performed a Fisher's exact test (Benjamini‐Hochberg adjusted *p* < 0.05) using topGO (v.2.48.0; Alexa and Rahnenführer 2009; node size = 20, “weight01” algorithm) using genes overlapping outlier windows identified through the XP‐*n*SL analysis. We then performed independent tests for each GO category (‘biological process’, ‘cellular component’ and ‘molecular function’). Scripts for this species analysis were developed using scripts previously developed by Colgan et al. (2019) and Colgan et al. (2022).

## 3 Results

### 3.1 Population Structure‐Based Analyses Identify Genetic Differentiation Between Island Populations

**FIGURE 1:**
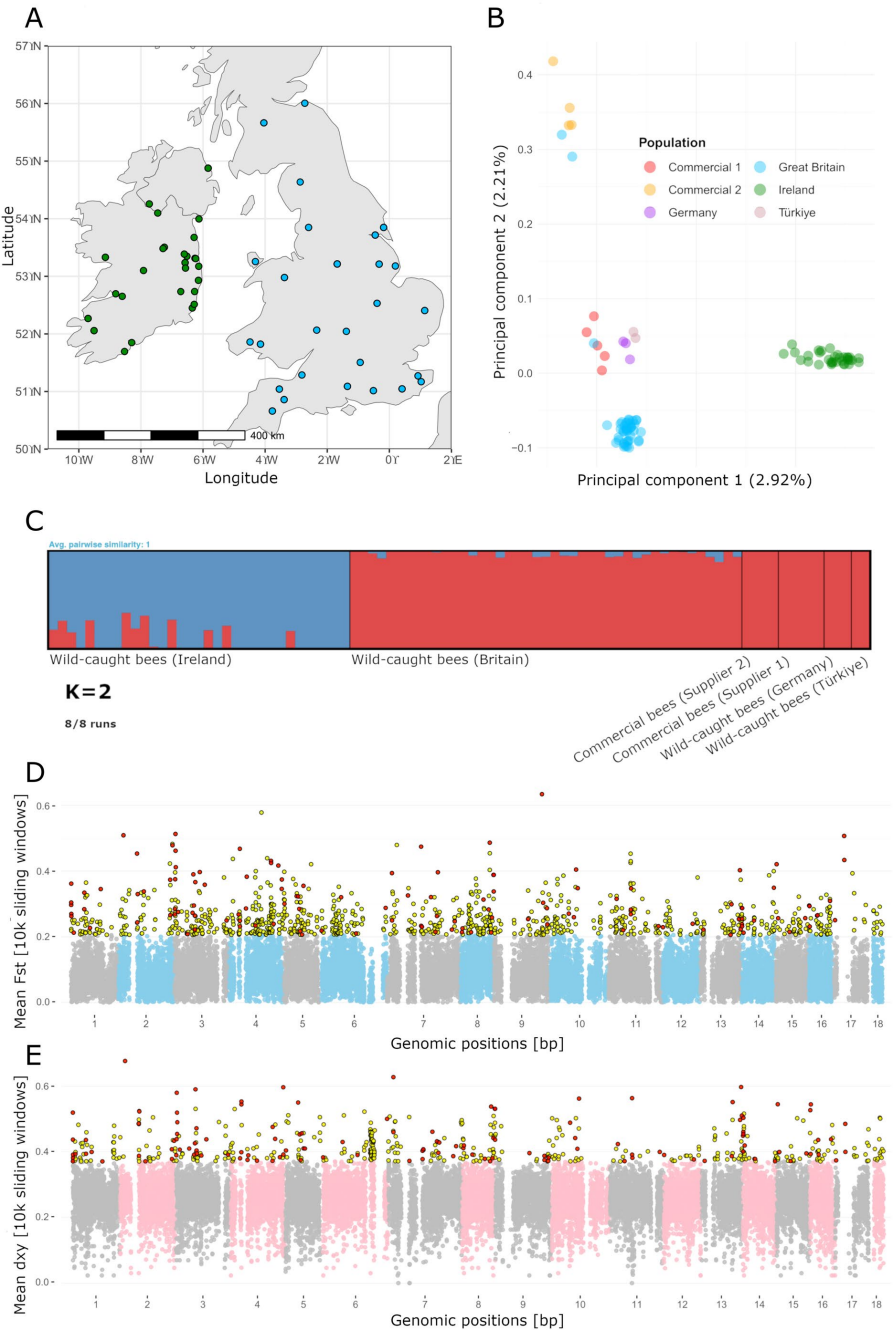
Population differentiation between *Bombus terrestris* collected in Ireland and Great Britain. (A) Map of collection sites of wild‐caught *B. terrestris* males previously sampled by Larragy et al. (2023) and Colgan et al. (2022), respectively. Each point represents a single collection site while color corresponds to island of sampling (green = Ireland; blue = Great Britain). (B) Scatterplot displaying principal components 1 and 2 from a principal component analysis based on pruned‐SNPs. PC1 separates samples collected on the island of Ireland from the remaining samples while PC2 largely separates bees from one of the commercial suppliers, as well as two bees sampled from Great Britain. Each geographical location and commercial supplier are indicated by individual colors. (C) Stacked barchart displaying the proportion of co‐ancestry shared amongst individuals estimated from an admixture‐based analysis performed on genome‐wide pruned SNPs, which provided greatest support for *K* = 2 subpopulations present within samples included in the present analysis. Similar to the results of the PCA, additional support is provided for the separation of bees sampled in Ireland from both wild‐caught bumblebees from other European geographical locations, as well as commercially‐imported bees from two independent suppliers to the Irish market. However, the admixture‐based analysis also points to evidence of admixture within both the Irish, and to a lesser extent, British population. (D, E) Manhattan plots displaying mean F_ST_ and mean dxy estimates for 10 kb sliding windows identified across the 18 chromosomal scaffolds of the *B. terrestris* genome assembly, respectively. Yellow dots indicate windows found with both elevated F_ST_ or dxy (*z*‐score < −2*σ*; *p* < 0.05) while red dots indicate windows identified with elevated F_ST_ and dxy, respectively.

To assess genome‐wide differences amongst *B. terrestris* individuals sampled across Ireland and samples from other European populations, including Great Britain (Figure 1A), we performed a PCA using a pruned dataset of genome‐wide SNPs. This analysis revealed clear separation of *B. terrestris* individuals sampled in Ireland and the rest of the samples along principal component 1 (PC1; Figure 1B), accounting for 2.92% of the variance in the dataset. Three general clusters, which group closely along PC1, separated distinctly across PC2 (explaining 2.21% of variance in the dataset). These clusters were (1) the majority wild‐caught bees sampled from Britain, (2) bees sampled from Germany, Turkey, Supplier 1, and one British individual, and (3) bees sourced from Supplier 2, as well as two wild‐caught bumblebees sampled in Great Britain. This highlights that even amongst commercial lines of bees which derive from natural populations, there is still distinction from the majority sampled in the wild (Figure 1B). Within the cluster representing bees from Germany, Turkey, and those sourced from Supplier 1, there is a slight separation along PC1 for these three groups, with the British‐caught individual clustering among Supplier 1 samples. While commercial *B. t. audax* individuals did not cluster directly with either wild *B. t. audax* population, commercial samples from both suppliers, and particularly those from Supplier 1, clustered closer to the British samples than Irish (Figure 1B). An ADMIXTURE‐based analysis further supported the separation of bees sampled from Ireland and the other geographical regions, which based on cross‐validation error values, found most support for two populations (*K* = 2; Figure 1C). Considering the low sample sizes of commercial, German, and Turkish populations in this dataset, which could influence patterns observed, we include results of the same ADMIXTURE‐based analyses for additional measures of K (*K* = 3, *K* = 4 and *K* = 5; Figure S1). Regarding genetic diversity between the two island populations, we found that wild‐caught British bees contained, on average, higher genome‐wide nucleotide diversity compared to bees sampled in Ireland (Wilcoxon rank‐sum test: w = 153724700, *p* < 2.2e‐16).

### 3.2 Divergent Patterns in Recent Selection Acting on B. terrestris Populations in Ireland and Great Britain

As our population‐based analysis revealed population differentiation between Irish and British populations (Figure 1), we investigated genome‐wide regions of elevated divergence using a sliding windows‐based approach and found 1015 windows with elevated F_ST_ estimates (z‐score < −2*σ*; *p* < 0.05) between bees sampled in Ireland when compared to the wild‐caught British bees (Figure 1D; Table S2c). These results were also supported by a dxy‐based analysis (Table S2), which also found genome‐wide patterns (*n* = 518 windows; z‐score < −2*σ*; *p* < 0.05; Table S2b) of divergence between Irish and British populations (Figure 1E). A proportion of windows were identified by both analyses (*n* = 162 windows) with a weak but positive correlation between measures across the genome (Spearman's rank correlation: rho = 0.24, *p* < 2.2e‐16). Sliding windows for dxy and F_ST_ estimates for additional pairwise comparisons among all geographical and commercial populations are presented in Table S2d,e.

**FIGURE 2:**
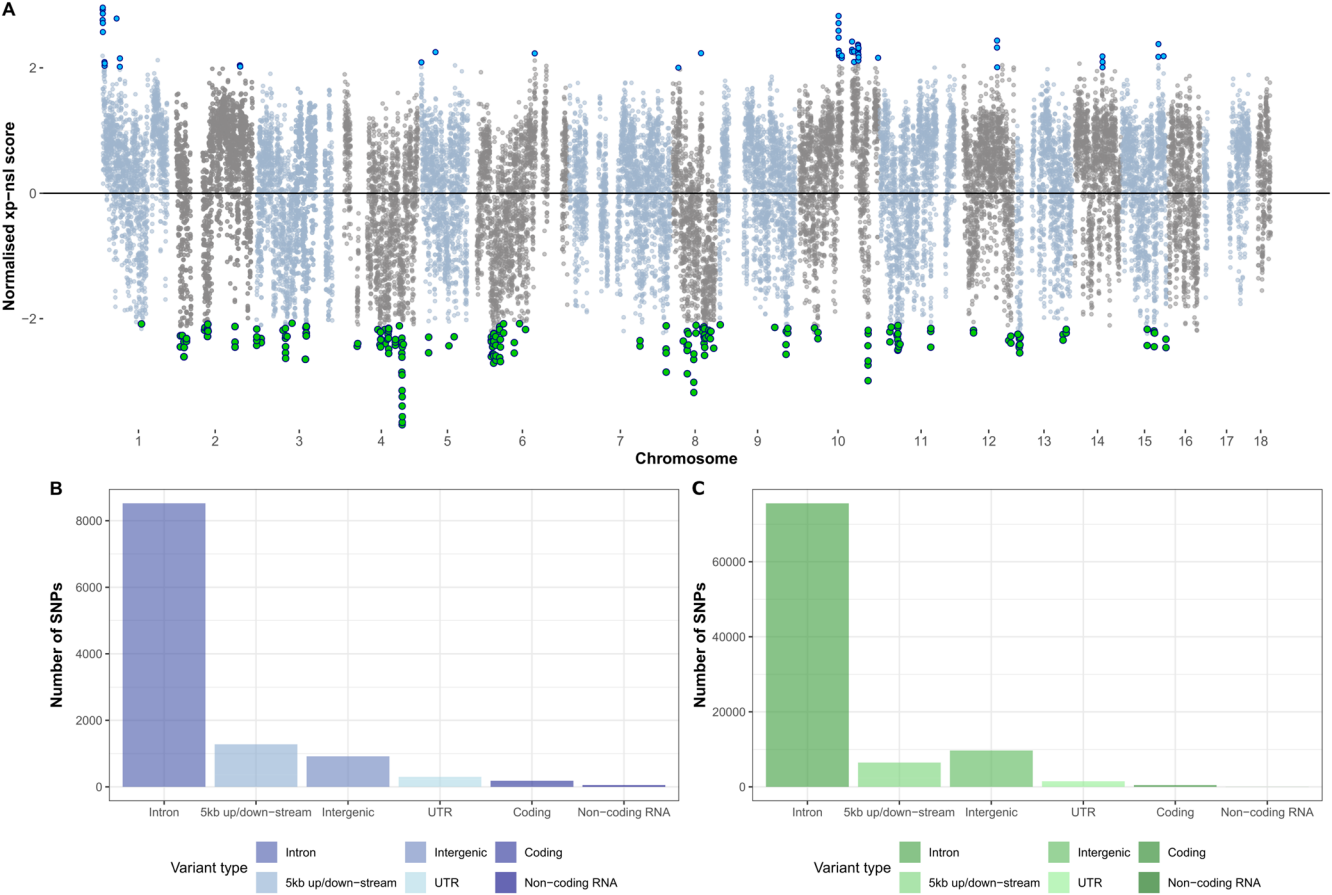
Divergent patterns of recent selection found in the genomes of wild‐caught bumblebees in Ireland and Great Britain. (A) Manhattan plot of mean normalized XP‐*n*SL scores assigned to polymorphic sites (SNPs) located within 10 kb sliding windows identified across the 18 chromosomal scaffolds of the *B. terrestris* genome assembly. Windows found on odd‐ and even‐numbered chromosomes are colored light blue and grey, respectively. Windows with significantly elevated measures of extended haplotype homozygosity (EHH; XP‐*n*SL scores; *z*‐score < −2*σ*; *p* < 0.05) are indicated in the British population (blue circles) and Irish population (green circles), respectively. Barcharts displaying the abundance of the main variant types assigned to SNPs found in windows with signatures of elevated EHH for the British (B) and Irish populations (C).

Further to this, we investigated signatures of recent or ongoing positive selection between *B. terrestris* populations by performing a cross‐population extended haplotype homozygosity‐based (XP‐*n*SL) analysis. Using normalized XP‐*n*SL scores, we found outlier windows with greater proportions of SNPs with signatures of positive selection (normalized |XP‐*n*SL| ≥ 2; *z*‐score < −2*σ*; *p* < 0.05; Table S3b) that were divergent between the Irish and British populations (Ireland: *n* = 241 windows; mean normalized XP‐*n*SL = −2.37, Great Britain: *n* = 56 windows; mean normalized XP‐*n*SL = 2.31, Figure 2A; Tables S3c–f) with significantly more windows displaying patterns of recent or ongoing selection identified in the Irish population (Binomial exact test: *p* < 2.2e‐16; 81% of outlier windows with evidence of selection). Merging neighboring windows revealed that the largest region under selection within the Irish population was present on chromosome four—a 110 kb region consisting of two genes (LOC100644394: POU domain, class 2, transcription factor 2; LOC125385041: terminal nucleotidyltransferase 5A‐like), with a mean normalized XP‐*n*SL = −3.69; (Figure 2A; Table S3e–f). In comparison, the largest region under selection in the British population was located on chromosome one, which was a 70 kb region consisting of 12 genes with the strongest signature present in a window overlapping a putative non‐coding RNA (LOC125384641) and a rabconnectin‐3B gene (LOC100650754: mean normalized XP‐*n*SL = 2.96; Figure 2A; Table S3c,d).

For both Irish and British populations, we identified genes overlapping outlier windows (British population: 144 genes; Irish population: 272 genes) and performed functional enrichment analyses finding enrichment of six GO terms within sites under selection in the Irish population (Fisher's exact test: BH‐adjusted *p* < 0.05), which were annotated with roles in transcription (‘positive regulation of transcription by RNA polymerase II’), signalling (‘cell–cell signalling’), and neurology (‘regulation of neurogenesis’) (Table S3g). In contrast, we found no significantly enriched GO terms (Fisher's exact test: BH‐adjusted *p* > 0.05) within genes found in regions of putative positive selection within the British population.

As an additional approach, we compared the proportion of variant types present in regions of ongoing selection between Irish and British populations. While overall more polymorphic sites were found in regions with signatures of selection in the Irish population, within both populations, intronic SNPs were the most common variant type (Ireland: 80% of all SNPs in outlier windows; Great Britain: 75% of SNPs in outlier windows; Figure 2B,C). In comparison, the proportion of other variant types differed between the two populations, with genomic regions under selection in the Irish population containing relatively more intronic and intergenic SNPs but fewer SNPs in upstream/downstream sites of genic regions, untranslated regions (UTRs), and coding regions compared to the British population (Chi‐square tests: *p* < 2.2e‐16; Figure 2B,C).

**FIGURE 3:**
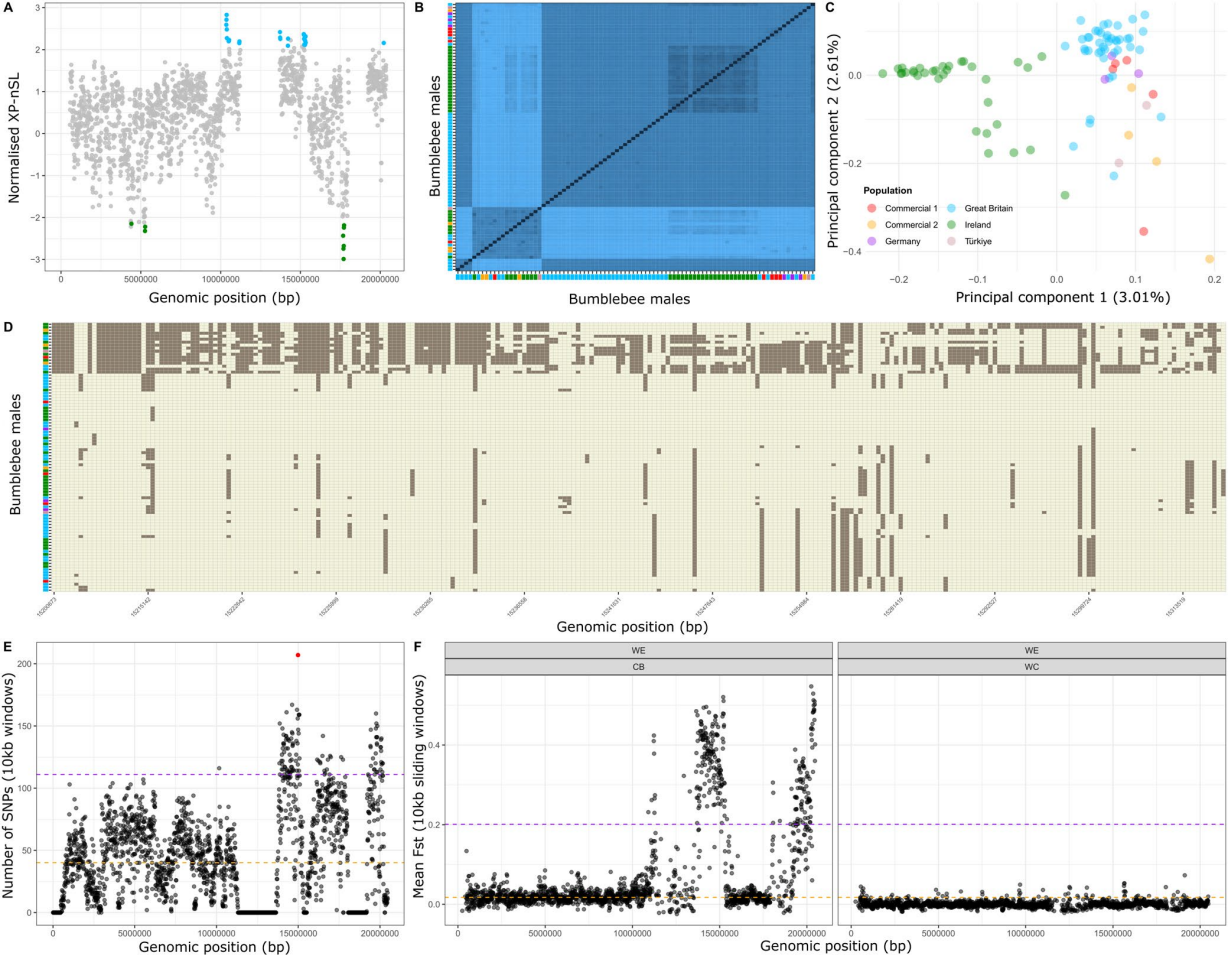
Region of elevated diversity under divergent selection on chromosome 10. (A) Windows‐based outlier analysis of genomic regions with elevated signatures of extended haplotype homozygosity as measured in XP‐*n*SL highlighted a region on chromosome 10 with signatures of recent selection in the British population. (B) Hierarchical clustering of samples based on polymorphic sites identified on chromosome 10 revealed the presence of multiple haplotypes present across European populations of *B. terrestis* subspecies, including *B. t. audax* (Ireland, Great Britain), *B. t. terrestris* (Germany), and *B. t. dalmatinus* (Türkiye). (C) Principal component analysis based on pruned SNPs also revealed closer clustering on chromosome 10 between Irish and non‐Irish bees. (D) Heatmap displaying a zoomed in section on the window with the highest XP‐*n*SL score with each row representing an individual bumblebee male and each column displaying the allele carried at a polymorphic site by an individual (tan = reference allele; brown = alternate allele). (E) Across all male samples, chromosome 10 contained windows with elevated number of SNPs (purple dashed line = top 1st percentile; orange dashed line = genome‐wide mean number of SNPs per 10 kb window), including the window with the highest number of polymorphic sites (red point) across the genome. (F) Scatterplots displaying mean F_ST_ per 10 kb sliding window calculated for a diploid dataset generated from wild (WE = wild‐caught *B. terrestris* collected in proximity to greenhouses; WC = wild *B. terrestris* sampled from independent sites not in proximity to agricultural lands) and commercial (CB) *B. terrestris* workers across chromosome 10 displaying genomic regions of elevated differentiation (purple dashed line = top 1st percentile; orange dashed line = genome‐wide mean F_ST_ per 10 kb window) between wild and commercial but not between wild‐caught samples collected from different sites in Sweden (data generated by Kardum Hjort et al. 2022).

A closer examination of genomic outliers with signatures of ongoing selection in both populations revealed a large polymorphic region on chromosome 10 (Figure 3A). Hierarchical clustering of all analyzed bees for chromosome 10 revealed evidence of shared haplotypes between wild‐caught and commercial bees (Figure 3B), which localized to a region on the latter half of chromosome 10. Similarly, a PCA performed for SNPs identified on chromosome 10 also found that certain wild‐caught Irish bees share greater genetic similarity to non‐Irish bees on this chromosome (Figure 3C). The presence of shared haplotypes between Irish wild‐caught and non‐Irish bees was further confirmed through the visualization of linked SNPs within the 10 kb window under strongest selection as identified based on our XP‐*n*SL‐based analysis (Figure 3D). In addition, a wider examination of SNP density on chromosome 10 revealed a neighboring window within close proximity with the highest number of SNPs (*n* = 207; Figure 3E) identified across all wild‐caught and commercial bees, which was four times higher than the genome‐wide mean (*n* = 48 SNPs per window). Furthermore, chromosome 10 contained more windows in the 1st percentile of windows based on SNP density (41% of windows in the top 1%), which was more than expected by random chance (Chi‐square test: *χ*2 = 458.65, df = 1, *p* < 2.2e‐16; Figure 3E).

To determine if patterns on chromosome 10 were independent of our analysis, we examined a second dataset from a previous study that sequenced wild‐caught *B. terrestris* workers collected in proximity to greenhouses (WE), wild *B. terrestris* sampled from independent sites not in proximity to agricultural lands (WC), and commercial *B. terrestris* (CB; Kardum Hjort et al. 2022). Using these data aligned against the same reference genome assembly, we found that the same regions on chromosome 10 were highly differentiated between wild‐caught and commercial bees (Figure 3F). For example, 73% of 10 kb windows located within the 1^st^ percentile based on mean F_ST_ were found on chromosome 10 when comparing each set of wild‐caught bees with commercials (mean F_ST_ WC‐CB: 0.384; mean F_ST_: WE‐CB: 0.332), which was more than expected by chance (Chi‐square test: *χ*2 = 2548.4, df = 1, *p* < 2.2e‐16). In contrast, we did not find the same pattern when comparing the two wild‐caught groups to each other, with measures of genetic differentiation across all of chromosome 10 over 100 times lower for comparisons between wild subgroups (mean F_ST_: WE‐WC: 0.00046) than for comparisons of each wild bee to the commercials (mean F_ST_ per pairwise comparisons for chromosome 10: WC‐CB: 0.09; WE‐CB: 0.076; Figure 3F).

## 4 Discussion

Geographically separated populations of bumblebee species face many environmental stressors, including competition, predation, and infection, as well as pressure from human‐related changes to their environments (Alford 1975; Colgan et al. 2022; Goulson et al. 2008; Larragy et al. 2023; Schmid‐Hempel 1995). Given the growing concerns over pollinator population declines at regional and global levels, it is increasingly critical to understand intraspecific variation, especially in cases where potentially vulnerable populations may be threatened by certain anthropogenic activities. A particularly relevant case is that of the island populations of *B. t. audax* bumblebees in Ireland and Great Britain. Despite being isolated for more than 14,000 years, Irish and British populations are classified as the same subspecies, a fact that has justified the generalized import and usage of *B. t. audax* commercial bumblebees across both islands. In their study, Franchini et al. (2023) provide evidence for genomic differentiation between British and commercial *B. t. audax*. However, knowledge of the extent of genomic differentiation between Irish and British *B. t. audax* populations, and indeed Irish and commercial *B. t. audax*, is currently limited, which has direct and indirect applications with respect to commercial agriculture, as well as conservation of natural genetic resources.

To address this gap in our understanding, we employed a population genomic approach and found clear evidence of strong genome‐wide differentiation between wild‐caught Irish and British populations of *B. t. audax*. In addition, we find evidence that both populations contain divergent signatures of recent positive selection, potentially driven by location‐specific pressures, that have targeted genes associated with the nervous system, transcriptional regulation, and cell‐signaling. Our analysis also identified a region under strong positive selection in the wild British population, which is highly polymorphic across wild‐caught European and commercial populations and centers on a vesicular acetylcholine transporter. We find evidence consistent with admixture between Irish and non‐Irish bees, particularly within a region on chromosome 10, a pattern also found by Kardum Hjort et al. (2022). Given the strong levels of genetic differentiation between Irish and non‐Irish bees, we discuss the potential implications of our findings on policy and management of commercial bumblebee colonies within island populations.

### 4.1 A Genetically Distinct Population of Bombus terrestris in Ireland With Evidence of Limited Admixture

Our study supports the existence of a genetically distinct population of *B. t. audax* on the island of Ireland, which expands upon previous genetic studies (Moreira et al. 2015) by demonstrating extensive genome‐wide differences between *B. t*. *audax* populations found in Great Britain and Ireland. Population‐level distinctions have frequently been reported for Irish insect species with wider‐European distribution such as the honeybee *Apis mellifera* (Hassett et al. 2018), the caddisfly *Plectrocnemia conspersa* (Wilcock et al. 2001) and the hairy wood ant ( *Formica lugubris* ; Mäki‐Petäys and Breen 2007). Indeed, the characterization of the native Irish population of honeybees (*A. m. mellifera*) has opened the door for its treatment as an individual conservation unit, distinct from non‐native and commercial hybrid populations. As a result, researchers and beekeepers in Ireland are advocating for the protection of the native honeybee and for stricter control on non‐native imports (Browne et al. 2020). The distinctions observed in many Irish fauna may be a consequence of the physical barriers, such as the Irish Sea, to restrict gene flow (Hassett et al. 2018; Moreira et al. 2015), or possibly the extended geographical separation from mainland Europe and potential isolation within refugia in Southwestern Ireland during the last ice age (Edwards and Brooks 2008; Teacher et al. 2009).

Our finding of genetic differentiation between Irish and British bees has implications also for commercial bumblebee use in Ireland given that *B. t. audax* imported into Ireland likely derives from British stocks, as supported by patterns highlighted in this study across two common commercial suppliers. While our sampling and sequencing of bees from independent commercial colonies used small sample sizes, they reveal patterns potentially consistent with high relatedness which adds to concerns of alleles being introduced into the natural landscape. Moreover, based on PCA, we find close clustering of three wild‐caught British bees (likely commercial escapees) with bees from commercial suppliers, despite the sampling of the wild‐caught British bees by Colgan et al. (2022) occurring in a different country and island, at least 5 years prior. With the exception of these samples, we found that most wild‐caught British individuals clustered separately from either group of commercially supplied bees, supporting the findings of Franchini et al. (2023) of distinctions between wild British and commercial *B. t. audax* populations.

Furthermore, we found evidence of limited admixture within the Irish *B. terrestris* population with other, non‐Irish *B. terrestris* , particularly evident within a region on chromosome 10. Interestingly, Kardum Hjort et al. (2022) also found a similar pattern, albeit truncated due to the use of an older assembly, on chromosome 10 which showed elevated distinctions between commercial and wild‐caught *B. terrestris* worker bees in Sweden. It is possible that the co‐ancestry we find between Irish and non‐Irish genomes may be a remnant of long‐standing genetic variation shared across bumblebee populations, as bumblebees are known to disperse over large distances, including across bodies of water (Bryant 1997, as cited in Kardum Hjort et al. 2023; Estoup et al. 1996; Fijen 2021; Moreira et al. 2015). Considering this, natural migration from Great Britain or nearby islands, such as the Isle of Man, to Ireland (distance of ~30 km at its shortest point between Ireland and Great Britain) is theoretically possible. However, rates of migration by *B. terrestris* from Ireland to Great Britain are predicted to be low (Moreira et al. 2015).

An alternative route to admixture within the Irish population may be linked to the importation into Ireland of commercial *B. terrestris* colonies—either the previously imported non‐native *B. t. dalmatinus* subspecies (Murray et al. 2013) or the currently imported *B. t. audax* subspecies, which may have been derived from British stocks. Indeed, our analysis using representative individuals collected from two common commercial suppliers demonstrates that such bees are more genetically similar to British or other European wild‐caught bees. Interestingly, of the wild‐caught Irish bees with elevated levels of co‐ancestry, many were sampled from eastern counties in Ireland, where commercial colony imports are concentrated for fruit production (Murray et al. 2008). Therefore, observed admixture within the Irish population may be the product of commercial introgression, as the escape of bumblebees from commercial colonies has been globally well‐documented (Dafni et al. 2010; Inoue et al. 2008; Schmid‐Hempel et al. 2014) and has occurred in other island ecosystems (Matsumura et al. 2004). Furthermore, while several studies do not find evidence of introgression between wild and commercial bumblebees (Hart et al. 2021; Kardum Hjort et al. 2022), others have reported admixture to varying degrees (Cejas et al. 2020; Franchini et al. 2023; Kraus et al. 2011; Seabra et al. 2019; Teagasc 2012). Admixture between native and introduced, non‐native populations is generally considered to impact the conservation status of the native species (Rosinger et al. 2021) and can have negative impacts (e.g., outbreeding depression) on the general fitness and adaptive ability of a population, even if limited (Allendorf et al. 2001; Franchini et al. 2023; Rhymer and Simberloff 1996).

### 4.2 Differential Signatures of Selection in Geographically Separated Populations of Wild *B. t.* Audax

Whole genome resequencing expands upon traditional population genetic methods by examining genetic differentiation between populations at a genome‐wide scale. Moreover, it can provide direct information on the targets of selection and the extent to which it is acting on a population, elucidating the adaptive potential of natural populations (Abondio et al. 2022; Eizaguirre and Baltazar‐Soares 2014; Vatsiou et al. 2016). Here, within the same subspecies, we find multiple genomic regions with divergent patterns of positive selection suggestive of differential selective processes acting on genes that are involved in fundamental physiological and developmental processes. While differences between *B. t. audax* sampled from Great Britain and Ireland may reflect differential responses to abiotic conditions that differ between the two islands, such as climate (e.g., Ireland experiences less temperature extremes and has slightly higher precipitation rates), biotic interactions (e.g., diversity and abundance of beneficial floral resources, and antagonistic competitors, parasites and disease—Ireland has a lower species richness than Great Britain; Harrison 2014), and human activity (e.g., land use and management—Ireland is dominated by agricultural grasslands for grazing livestock; Central Statistics Office 2021). These genome‐wide differences in terms of genetic differentiation and associated signatures of selection within the same subspecies highlight the need for greater biomonitoring of island populations to determine genetic variation and potential risks from environmental pressures.

Like other species, wild bees are experiencing ever‐increasing changes to their environments caused by human‐related activities such as agriculture and urbanisation (Dudley and Alexander 2017; Grimm et al. 2008). To understand further differences between Irish and British populations, we detailed genomic regions under selection in each island population, finding genes with putative roles in development, transcriptional regulation, and the functioning and development of the nervous system. Regions with the strongest signatures of recent adaptation included potential targets of pesticides, including a vesicular acetylcholine transporter, which was the most highly polymorphic region across *B. terrestris* at the European level. Homologues of this gene have been described to function in the catalysis of the neurotransmitter acetylcholine (Liu et al. 2022), identifying the gene as a potential target of insecticides. While this specific gene has not been functionally assessed in bumblebees, nor has its expression profile been described as affected by exposure of bumblebees to common pesticide classes, such as neonicotinoids (Bebane et al. 2019; Colgan et al. 2019), the extent of polymorphism at the regional level and its putative role in insecticide susceptibility (Goodchild et al. 2024; Liu et al. 2022; Sluder et al. 2012) make it an interesting case for future study. Furthermore, the region should also be noted for the presence of haplotypes within both wild‐caught and commercial bumblebees (Figure 3). While such patterns may be the result of long‐standing genetic variation shared across European populations, introgression from commercial bees, which is a topic of ongoing concern, may also contribute to patterns we see here. Future studies incorporating greater numbers of wild‐caught and commercial bees across a wider European scale will provide greater insight into genetic variation maintained at the species level, allowing for greater ability to determine the source of these patterns.

In addition to the putative vesicular acetylcholine transporter, other targets of insecticides were identified in regions of extended haplotype homozygosity within the Irish population. These genes encode proteins that serve as receptors (LOC100648987: *acetylcholine receptor subunit alpha*; LOC100643486: *NMDA receptor 2*) for two key excitatory neurotransmitters, acetylcholine and glutamate, respectively, which act within the insect brain (Tomizawa and Casida 2001) and are implicated in processes key for olfactory learning and memory formation in insects (Cartereau et al. 2022; Gauthier and Grünewald 2012). However, they have also been identified as targets of common insecticides (Li et al. 2023; Moffat et al. 2016). As the mode of action of many insecticides is to selectively target and disrupt insect neurotransmission (Vehovszky et al. 2015; Zhao et al. 2004), changes to genes relating to these processes could contribute to an adaptive response against abiotic stressors, such as insecticide use, or a more general role in detoxification, within the Irish population.

Developmental genes also displayed strong signatures of positive selection in the Irish population. As processes relating to embryo and larval development are usually under purifying selection due to their essential nature (e.g., Lawrie et al. 2013), it may be considered surprising to see strong signatures of differential selection between Irish and British *B. t. audax* in genes putatively involved in neurogenesis. However, as normal development of anatomical structures in embryonic and larval stages is vital to the establishment of functioning organs and physiological processes that are essential to adult survival (Campos and Hartenstein 1985; Wang et al. 2015), changes in the local environment representing new risks or challenges may be reflected in changes in or close‐by developmental genes. In addition, the nervous system is essential for coordinating responses and associated behaviors that allow organisms to process environmental stimuli, which is reflected in numerous population genomic studies of insects identifying strong signatures of positive selection acting on neurological genes (Colgan et al. 2022; Harpur et al. 2014; Lange et al. 2022). In the case of bees, coordinated behavioral displays are essential in assessing food or nesting resources, communicating with nestmates, as well as foraging in complex landscapes (Klowden 2013; Nation 2008). Considering the continued anthropogenic‐driven change to pollinator habitats (Millard et al. 2021), our findings may indicate that bumblebee nervous systems and social behaviors may be under strong selection pressure to cope with novel or altered stressors.

### 4.3 Applications of Our Findings

Our findings reinforce the utility and value of population genomic approaches in the biomonitoring of natural populations, especially those on islands, to identify distinctive populations that are potentially locally adapted and/or vulnerable. While our findings greatly contribute to and expand upon the classification of a distinct *B. t. audax* population on the island of Ireland (Moreira et al. 2015), we believe our approach emphasizes more broadly the necessity of evaluating island populations to inform their conservation as natural resources. Conserving genetic variability within the natural populations of bumblebees is essential for the genetic health and adaptability of these insects across their range and will also ensure the future viability of captive‐bred bumblebee lines for pollination services. This study also highlights the importance of taking caution when generalizing about the biodiversity of island systems, as distinctive subpopulations often exist within these (Hassett et al. 2018; Sleeman 2014) which, as suggested by Rhymer and Simberloff (1996), require accurate taxonomic descriptions. The genetic differentiation of wild‐caught and commercial bees revealed by this study emphasizes the need for risk‐mitigating actions in the management of imported colonies on‐site, such as the use of queen excluders and proper destruction of colonies at the end of the crop flowering period. Based on our findings, we would also encourage commercial breeders to explore the possibility of establishing lines of captive *B. terrestris* colonies (e.g., through supplementation with wild‐caught individuals) that are genetically representative of the distinct *B. terrestris* subpopulations found on each of the islands of Ireland and Great Britain, as has been performed successfully elsewhere, such as in the Canary Islands and Sardinia (Velthuis and van Doorn 2006). We believe our study calls attention to the importance of understanding intraspecific variation and adaptation in wild pollinators to inform management practices and local conservation efforts aiming to conserve these beneficial organisms.

## Conflicts of Interest

The authors declare no conflicts of interest.

## Supporting information


**Data S1:** Supporting information.

**Data S2:** Supporting information.

**Data S3:** Supporting information.

**Data S4:** Supporting information.

**Data S5:** Supporting information.

## Data Availability

Raw sequencing data have been uploaded and are publicly available from the NCBI Sequence Read Archive (BioProject Accession: PRJNA1095208). This study also used publicly available datasets listed under BioProjects PRJNA870415 (Larragy et al. 2023) and PRJNA628944 (Colgan et al. 2022). Scripts used in the present analysis are included as supplemental information and will be publicly hosted for reuse on: https://github.com/Joscolgan/bter_island_comparison.
