# Supplementary material for: A Genome‐Wide Analysis of Structure and Evolution in Irish and British Populations of Bombus terrestris (L. 1758): Implications for Genetic Resource Conservation
Source: Evol Appl. 2025 Aug 8;18(8):e70141. doi: 10.1111/eva.70141 (PMC12334553; doi:10.1111/eva.70141)
Supplement: Supplementary file 1 — Data S1: Supporting information. [file EVA-18-e70141-s003.docx]

**Supplemental information**

**
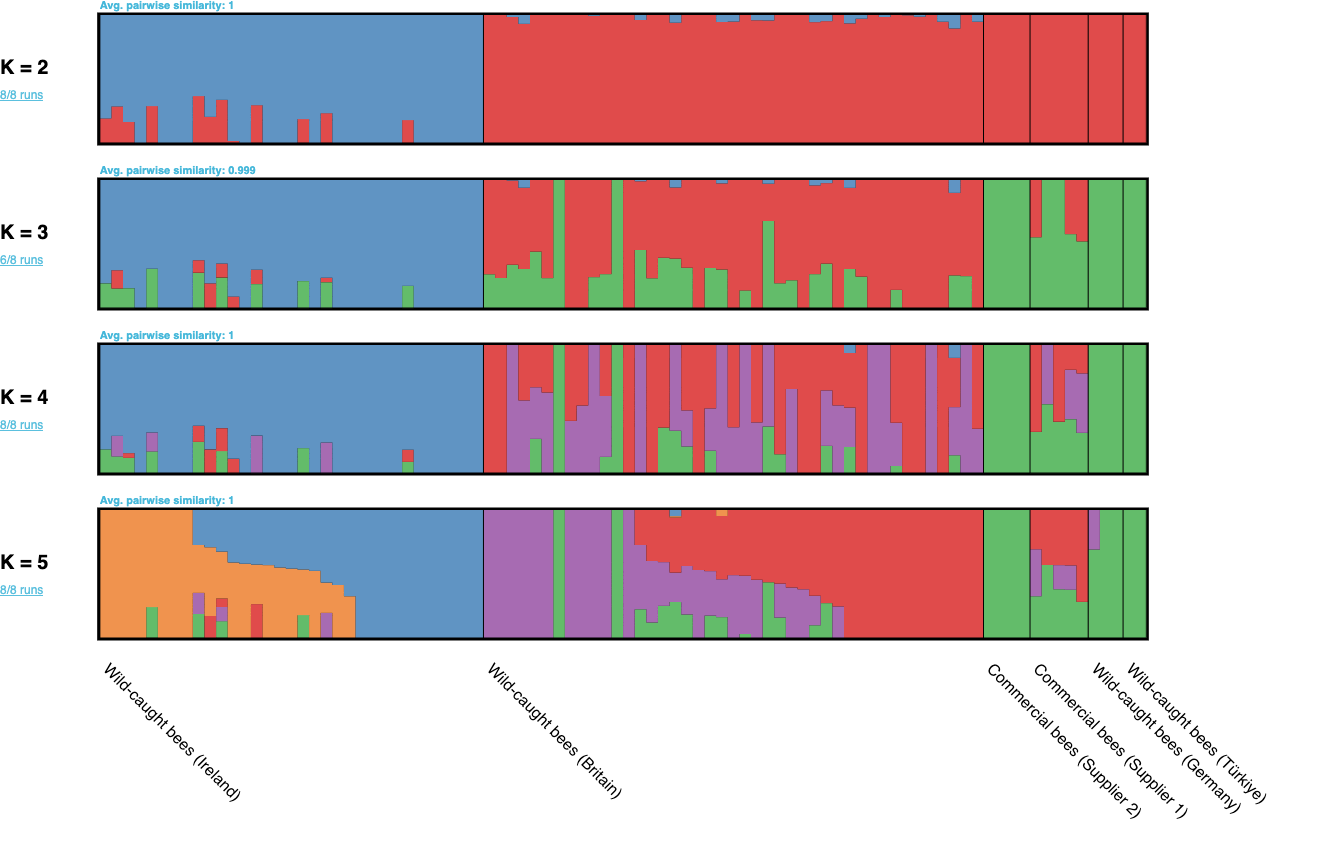
**

**Figure S1** **Results of ADMIXTURE-based analyses for additional K values.** Stacked barcharts for K = 2, K = 3, K = 4 and K =5 displaying the proportion of co-ancestry shared amongst individuals assessed using admixture-based analysis on genome-wide (pruned) SNPs. Greatest support was found for *K* = 2 subpopulations in the datasets analysed.
